# Processing and Thermal Response of Temperature-Sensitive-Gel(TSG)/Polymer Composites

**DOI:** 10.3390/polym10050486

**Published:** 2018-05-01

**Authors:** Jin Gong, Eiichi Hosaka, Kohei Sakai, Hiroshi Ito, Yoshikazu Shibata, Kosei Sato, Dai Nakanishi, Shinichiro Ishihara, Kazuhiro Hamada

**Affiliations:** 1 Department of Polymer Science & Engineering, Graduate School of Organic Materials Science, Yamagata University, 4-3-16 Jonan, Yonezawa, Yamagata 992-8510, Japan; txy33515@st.yamagata-u.ac.jp; 2 Department of Mechanical Systems Engineering, Graduate School of Science and Engineering, Yamagata University, 4-3-16 Jonan, Yonezawa, Yamagata 992-8510, Japan; tmf62483@st.yamagata-u.ac.jp; 3 Kohjin Film & Chemicals Co., Ltd., 1-1 Koukokumachi, Yatsushiro, Kumamoto 866-8686, Japan; y_shibata@kohjin.co.jp (Y.S.); k_sato@kohjin.co.jp (K.S.); d_nakanishi@kohjin.co.jp (D.N.); s_ishihara@kohjin.co.jp (S.I.); kazuhiro_hamada@kohjin.co.jp (K.H.)

**Keywords:** temperature sensitive, gel, controllable gas permeability, breathable film, polymer composite, processing

## Abstract

Temperature-sensitive gels (TSGs) are generally used in the fields of medical, robotics, MEMS, and also in daily life. In this paper, we synthesized a novel TSG with good thermal durability and a lower melting temperature below 60 °C. We discussed the physical properties of he TSG and found it provided excellent thermal expansion. Therefore, we proposed the usage of TSG to develop a strategic breathable film with controllable gas permeability. The TSG particles were prepared firstly and then blended with linear low-density polyethylene/calcium carbonate (LLDPE/CaCO_3_) composite to develop microporous TSG/LLDPE/CaCO_3_ films. We investigated the morphology, thermal, and mechanical properties of TSG/LLDPE/CaCO_3_ composite films. The film characterization was conducted by gas permeability testing and demonstration temperature control experiments. The uniformly porous structure and the pore size in the range of 5–40 μm for the TSG/LLDPE/CaCO_3_ composite films were indicated by SEM micrographs. The demonstration temperature control experiments clearly proved the effect of the controllable gas permeability of the TSG and, more promisingly, the great practical value and application prospects of this strategic effect for the temperature-sensitive breathable film was proved.

## 1. Introduction

Gels are a promising material having good compatibility with biological tissues. The three-dimensional cross-linked network of the gel, like collagen in tissues, is the framework structure contributing to the mechanical strength. Temperature-sensitive gels (TSGs) exhibit physical changes in response to changes in temperature. One research field in which the authors are interested is the synthesis and application of high-performance crystalline gels. Crystalline gels are gels with high crystallinity polymerized by introducing crystalline side-chains into the three-dimensional structure. Many crystalline gels have been synthesized [1,2,3,4,5,6,7]. TSGs are a kind of crystalline gel, and they are expected to apply in the fields of medical, robotics, as well as daily life [1,5,8]. The melting of side-chain crystals leads to the changing behavior in the physical properties, especially in viscosity, volume, and mechanical strength. The physical property changes provide the temperature sensitivity of TSGs. TSGs become viscous liquids and change their volume at the transfer temperature (melting temperature) [3,4,7]. Generally, volume change, especially thermal expansion, is considered one of the major drawbacks in polymer applications. Many researchers have been placing a great deal of effort in modifying the thermal expansion of polymers and polymer composites [9,10,11,12,13,14,15,16]. Usually it is the other way around, so we propose to make full use of the thermal expansion inversely.

Functional porous membranes/films, which can control the transfer of air, humidity, light, and temperature, are important and have attracted much attention due to their wide use in fields such as food packaging, electronics, the energy sector, and daily necessities [17,18,19,20,21,22,23,24,25,26,27,28]. Most of these porous membranes/films are environment- or signal-responsive. Even though environment-responsive pore size adjustment of porous membranes/films is desirable, environment-responsive pore size regulation remains challenging. In this paper, we report a temperature-responsive pore film that achieves the opening/closing of pores. The temperature-responsive pore adjustable film was fabricated with hybrid materials of TSGs within LLDPE/CaCO_3_ composites. The TSG used here was designed to have high thermal expansion.

## 2. Materials and Methods

### 2.1. Materials

Two monomers containing vinyl groups (–CH=CH_2_) were used as monomers. One monomer (M1) consists of a vinyl group and a long alkane chain with more than sixteen methylene units. The other monomer (M2) has a pendent group of acetoxy (–COOCH_3_). *N,N*′-methylenebisacrylamide (MBAA) used as a cross-linker, and benzophenone (BP) used as a UV polymerization initiator were obtained from Wako Pure Chemical Industries Ltd., Osaka, Japan. LLDPE/CaCO_3_ composite were supplied by Kohjin Film and Chemicals Co., Ltd., Yatsushiro, Japan. All the above chemicals are used as received.

### 2.2. Preparation of TSG Particles

Firstly, the TSG sheets are synthesized via bulk photopolymerization. The synthesis procedure is as follows: Into a 50 mL snap vial were placed monomer M1, monomer M2, and cross-linker MBAA. The mixture was stirred for 30 min and then initiator BP was added. The molar ratio of M1: M2: MBAA: BP was 1.00:3.00:0.02:0.04. The nitrogen bubbling was performed during the above process to eliminate the oxygen, which prevents the radical polymerization. Then the solution was poured into a self-made mold and irradiated under a UV lamp at 30 °C for 9–20 h. The obtained TSG sheets were frozen with liquid nitrogen and finely ground into particles using a dancing mill (ALM90DM, Nitto Kagaku Co., Ltd., Nagoya, Japan). The prepared TSG particles are a white powder.

### 2.3. Preparation of TSG Films

With these frozen, ground TSG particles, Uniform TSG films were prepared by a vacuum heating press machine (Imoto machinery Co., Ltd., Kyoto, Japan) for thermal mechanical analysis (TMA) testing. Hot pressing was conducted for 2 min at 140 °C under 8 MPa pressure between non-stick sheets. This ensured the complete melting/fusion of TSG particles during film formation. A force of 4 MPa was applied and maintained for 1 min during the cooling period until room temperature was reached. The thickness of the resultant TSG films was in the range of 200 to 300 μm.

### 2.4. Preparation of TSG/LLDPE/CaCO_3_ Composites and Films

The pellets of TSG/LLDPE/CaCO_3_ composites were prepared by a twin-screw extruder (LABO PLASTOMILL 4C150, Toyo Seiki Seisaku-sho, Ltd., Tokyo, Japan), set to a speed of screw rotation of 60 rpm and a speed of feeder rotation of 10 rpm. The extruder barrel temperature profile was 190/200/200 °C from the feeder to the die end. The mixing weight ratio of TSG to LLDPE/CaCO_3_ was 10 to 90 wt %.

The flat films of TSG/LLDPE/CaCO_3_ were extruded from the T-die twin-screw extruder (LABO PLASTOMILL MT100B type, 2D15W, Toyo Seiki Seisaku-sho, Ltd., Tokyo, Japan) using the resultant pellets, set to a speed of screw rotation of 9 rpm, a speed of feeder rotation of 10 rpm, and a temperature program of 190/200/200 °C. To further uniformize the film thickness, a vacuum heating press machine (Imoto machinery Co., Ltd., Kyoto, Japan) was used. Hot pressing was conducted at 200 °C for 1 min under 20 MPa pressure, then cooling pressing was conducted for 1 min under 10 MPa pressure at room temperature. The hot-pressed uniform films were used for biaxial stretching, and also punched out to produce dumbbell-shaped specimens for tensile testing.

### 2.5. Characterization

#### 2.5.1. Differential Scanning Calorimetry (DSC)

The melting and crystallization behavior of TSG particles were studied using a differential scanning calorimeter (DSC) (Q-200, TA instruments Japan Inc., Tokyo, USA) operating under a nitrogen flow. Samples of about 5 mg were heated to 70 °C at a heating rate of 10 °C/min and held for 1 min to eliminate the thermal prehistory. Next, samples were cooled to 10 °C at a cooling rate of 5 °C/min and held for 1 mi, and finally reheated again to 70 °C at the same rate. Premium hermetic pans (TA Instruments T zero #901683.901) were used for the measurements.

#### 2.5.2. Wide-Angle X-Ray Scattering (WAXS)

To determine the crystallinity of TSG particles, wide-angle X-ray scattering (WAXS) was performed on a X-ray diffractometer (Ultima IV, Rigaku Corporation, Akishima, Japan) with nickel-filtered Cu Kα radiation at a scanning rate of 5 °·min^−1^. The degree of crystallinity (*W_c_*, %) was evaluated according to the following formula reported by W. Ruland [29]:

(1) $$W_{C}=\;\frac{I_{c}}{I_{c\;}+I_{a}}\times 100$$

where *I_c_* and *I_a_* are the scattering intensities of crystalline region and amorphous region, respectively.

#### 2.5.3. Thermal Gravimetric Analysis (TGA)

The thermal stability of TSG particles was studied using a thermal gravimetric analysis (TGA) (Q-50, TA instruments Japan Inc., Tokyo, USA) at the heating rate of 10 °C/min from ambient temperature to 600 °C in a nitrogen atmosphere. The nitrogen flow rate was adjusted to 60 mL/min for the sample and 40 mL/min for the balance. The sample weight was more than 10 mg.

#### 2.5.4. Thermal Mechanical Analysis (TMA)

The dimensional properties of TSG, LLDPE/CaCO_3_, and TSG/LLDPE/CaCO_3_ were measured using a TMA (Q-400, TA instruments Japan Inc., Tokyo, USA) operated in expansion mode of film/fiber. The samples were heated, cooled, and held under isothermal conditions by varying the loading applied to the samples. TMA tests were run from 10 to 70 °C using specimens punched out from hot-pressed 200–300 μm thick films. Test specimens were strips 20 mm long and 2 mm wide. TMA tests were run under the following conditions with two cycles: isothermal at 10 °C for 5 min, 10–70 °C at 5 °C/min, heating process force: 0.02 N, isothermal at 70 °C for 10 min, 70–10 °C at 2 °C/min, cooling process force: 0.00 N.

The coefficient of linear thermal expansion (α) over a temperature range was calculated according to the well-known formula (2):

(2) $$\alpha =\frac{\Delta L}{L_{0}}\cdot \frac{1}{\Delta T}$$

where Δ*L* represents the elongation (change in length), *L_0_* represents the initial specimen length, and Δ*T* is the change in temperature [30].

#### 2.5.5. Pressure-Volume-Temperature (PVT) Test

The PVT tests were performed to measure the volume thermal expansion using a P-V-T test system (MPPS-1, Toyo Seiki Seisaku-sho, Ltd., Tokyo, Japan) under 1 MPa pressure. The cooling rate was 5 °C/min. In the case of TSG, the measurement was made in the 30–120 °C range, and with the LLDPE/CaCO_3_ composite, in the 30–200 °C range [31,32].

#### 2.5.6. Tensile Testing

The mechanical properties of the specimens were determined by tensile tests using a versatile tension-testing machine (STROGRAPH VGS-1-E, Toyo Seiki Seisaku-sho, Ltd., Tokyo, Japan). The specimens were dumbbell-shaped, punched out from hot-pressed 200–300 μm thick films, with dimensions of 35 mm × 6 mm × 2 mm (test specimen No. 7, JIS K6251 standard). The specimens were elongated at a constant rate of 300 mm/min until the specimens rupture at room temperature. The tensile strength at break, the Young’s modulus, and elongation at break were evaluated from the tensile stress-strain curves. Testing was run on a minimum of five specimens. The reported data were the average of the results of five specimens.

#### 2.5.7. Scanning Electron Microscope (SEM)

The morphology of TSG particles and LLDPE/CaCO_3_ and TSG/LLDPE/CaCO_3_ composite films was observed using a scanning electron microscope (SEM) (Miniscope TM-1000, Hitachi High-Technologies Corporation, Tokyo, Japan) at accelerating voltages of 10–15 kV. Samples were sputter-coated with platinum-palladium for SEM analyses and observed in a magnification range from 100× to 1000×. The sizes of TSG particles and the pores of microporous composite films were measured from the SEM micrographs. To obtain the mean size, at least 100–150 particles and pores were selected randomly.

#### 2.5.8. Air Permeability Measurement

The air permeability of the biaxially-oriented microporous LLDPE/CaCO_3_ and TSG/LLDPE/CaCO_3_ films was characterized through a Gurley-type densometer (G-B3C, Toyo Seiki Seisaku-sho, Ltd., Tokyo, Japan), according to JIS P 8117 and ISO 5635. The time (t) for a settled volume (100 mL) of air to pass through the sample with a fixed area (6.45 cm^2^) at 20–58 °C was measured. The temperature cycles were defined as follows: 20 °C, then 50 °C, and 58 °C to finally measure the air permeability at 20 °C. The temperature was progressively increased and controlled by an oven sensor placed on the flat module surface.

#### 2.5.9. Temperature Control Experiment

To verify the controllable air permeability of TSG/LLDPE/CaCO_3_ composite films, temperature control experiments were conducted using the handmade heating pouches by using the biaxially-oriented composite films as the outer layer. Inside the heating pouch, an iron mixture that was taken out of a commercial portable hand warmer was used. The main ingredients of the iron mixture are iron powder, a water absorbent material, and activated carbon [33]. The oxygen and water react with the iron powder located inside to form iron oxide and release heat. If the pouch admits more oxygen (air), the reaction occurs more quickly, eventually, the quick release of too much heat leads to the rapid rise in temperature. A temperature-measuring instrument (Kohjin Film and Chemicals Co., Ltd., Yatsushiro, Japan) was used to detect the temperature changes of the heating pouches with time.

## 3. Results and Discussion

### 3.1. Size, Morphology, and Thermal Properties of TSG Particles

**Figure 1:**
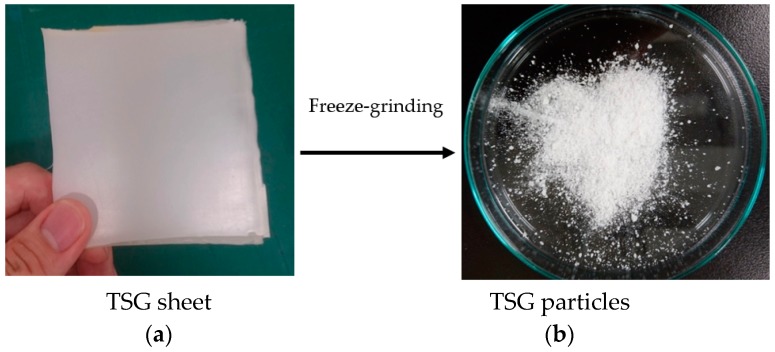
TSG sheet synthesized via bulk photopolymerization (**a**) and TSG particles prepared by freeze-grinding technique (**b**).

**Figure 2:**
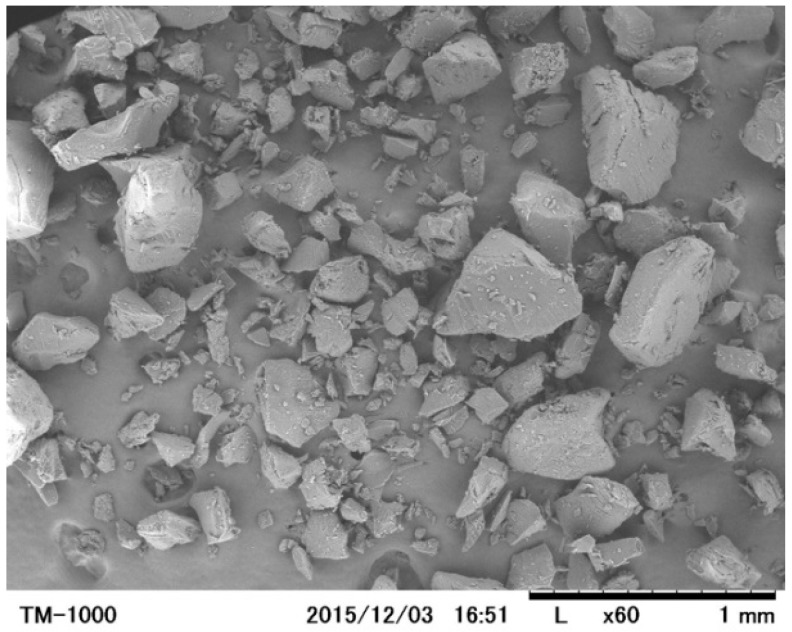
SEM micrograph of TSG particles. TSG particles have a size in the range of from 10 to 400 μm, and their shape is angular, like crushed stones.

**Figure 3:**
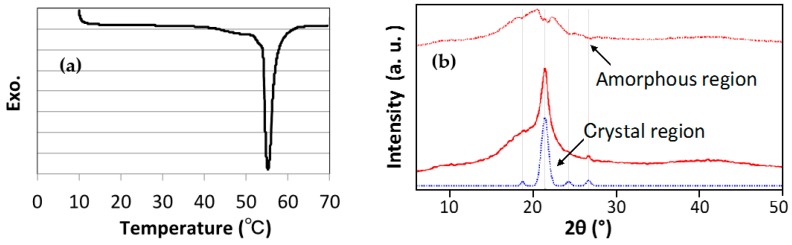
Differential scanning calorimetry (DSC) melting curve (**a**) and wide angle X-ray scattering (WAXS) (**b**) of TSG particles.

**Figure 4:**
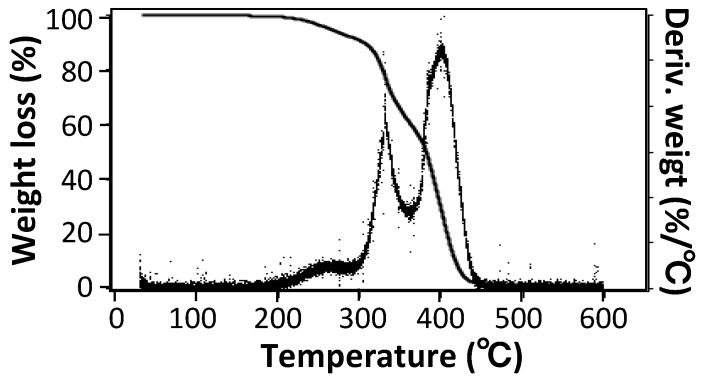
The weight loss vs. temperature plot and its derivative curve measured by thermogravimetric analysis (TGA) for TSG particles.

The synthesized TSG sheet and TSG particles made from frozen, ground TSG sheets are shown in Figure 1. The SEM image of the TSG particles is shown in Figure 2. It was found that TSG particles have a size in the range of 10 to 400 μm, and their shape is angular, like crushed stones. The DSC melting curve for TSG particles, registered during the second heating at a rate of 10 °C/min, is shown in Figure 3a. A clear single endothermal peak at 55.3 °C was observed, which belongs to the melting point of TSG crystallites. Figure 3b is the intensity vs. 2θ plot for TSG particles measured by wide-angle X-ray scattering (WAXS). The degree of crystallinity (*W_c_*) was estimated from the areas under the curves of the crystal region and amorphous region [29,34], and the value of *W_c_* was about 41%, indicating TSG particles have high crystallinity, and the crystals melt when increases the particles’ temperature to nearly 55.3 °C. Figure 4 shows the thermogravimetric analysis (TGA) of TSG particles. The TGA plot shows that the TSG particles undergo thermal degradation beginning at 206 °C and with a total mass loss of 99.3%. It also shows that the temperatures at which there was a mass loss of 10 wt % (*T*_10%_) and 50 wt % (*T*_50%_) are 305 °C and 381 °C, respectively. This suggests TSG particles have enough high thermal durability resistance to the general polymer processing temperature and are suitable for being used as functional fillers to develop polymer composites [3,35].

### 3.2. Linear and Volumue Thermal Expansion of TSG

**Figure 5:**
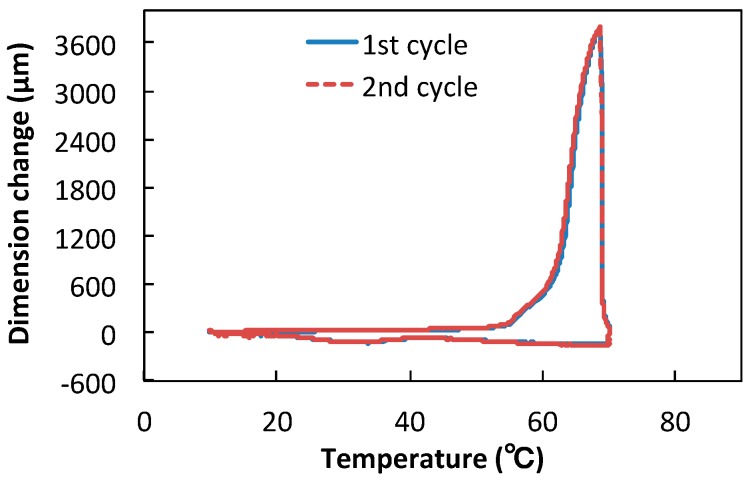
TMA curves of the dimensional change versus temperature obtained in the first cycle and second cycle for the TSG.

In Figure 5, the TMA expansion curve of the TSG is presented, showing the linear dimensional changes versus temperature. A sharp dimension increase appears between 50 and 60 °C, which is near the melting temperature of the TSG. The maximum value of the dimension change reached 3784.9 μm. The values of the linear coefficient of thermal expansion for the temperature range 10–70 °C are given in Table 1. When the temperature approaches 55 °C, which is in the vicinity of the melting point of TSG, α begins to raise, signaling the beginning of the crystal melting. When the melting point is approached, the molecular mobility of TSG chains increases and the glassy solid becomes a viscous liquid, thus α increases rapidly from 322.2 × 10^−5^/°C at 55–65 °C to 2817.0 × 10^−5^/°C at 65–70 °C. After the first cycle, the curve of the second cycle overlapped well, suggesting that the TSG has a good shape memory property. The rapid increase of α ensures the pores decrease in size, or partially close. Hence, introducing TSG particles into the LLDPE/CaCO_3_ composite having microporosity enables us to give the film pore-size adjustment function responding to the change in temperature. Furthermore, the shape memory property makes the reduced pores re-expand with decreasing temperature, which reveals that the pore-size adjustment function is reversible and hopeful to sustainable applications. 

The volume thermal expansion of TSG, and that of LLDPE/CaCO_3_ as a comparison, were measured by PVT testing. The volume was calculated from specific volume obtained from PVT results. The volumes and the change rates of volume for TSG and LLDPE/CaCO_3_ were summarized in Table 2. The change rate of the volume of the TSG is 3.5 times that of the LLDPE/CaCO_3_ composite. The high volume thermal expansion of the TSG near the melting temperature is critical to make the pores smaller to control the air permeability for the microporous TSG/LLDPE/CaCO_3_ composite films.

### 3.3. Effect of TSG on Mechanical Properties and Thermal Expansion

**Figure 6:**
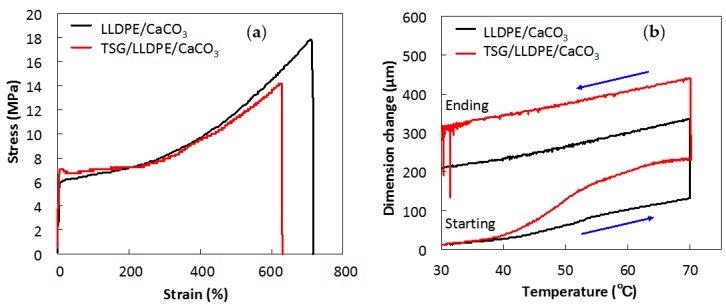
Tensile stress-strain curves (**a**) and dimension change as a function of temperature supplied by TMA testing (**b**) for LLDPE/CaCO_3_ and TSG/LLDPE/CaCO_3_ composites.

Tensile stress-strain curves of LLDPE/CaCO_3_ and TSG/LLDPE/CaCO_3_ are presented in Figure 6a. The average values of the mechanical properties are given in Table 3. The tensile strength at break, Young’s modulus, and elongation at break of TSG/LLDPE/CaCO_3_ composites and non-TSG-added LLDPE/CaCO_3_ composites were compared. The Young’s modulus was measured in the strain range of 0–0.04% in which the stress is proportional to the strain. Although a decrease in tensile strength at break was noted, the elongation of TSG/LLDPE/CaCO_3_ composites increased from 571% to 699%, while the Young’s modulus maintained almost the same value with the addition of 10 wt % TSG particles. The addition of TSG did not impair the balanced mechanical properties of the composites.

In Figure 6b, the TMA curves of LLDPE/CaCO_3_ and TSG/LLDPE/CaCO_3_ composites are presented. The dimension change is recorded as a function of temperature. When temperature increases to 40 °C, close to the melting point of the TSG, a rapid and large increase in dimension change occurred for TSG/LLDPE/CaCO_3_ composites, while for the non-TSG-added LLDPE/CaCO_3_ composites, the dimension change was not so obvious. The dimension change of TSG/LLDPE/CaCO_3_ composites at 70 °C reached 232.07 μm, which is 1.8 times that of LLDPE/CaCO_3_ composites (129.71 μm). The total dimension change after one heating/cooling cycle increased 1.5 times from 210.94 to 314.47 μm by adding the TSG. Namely, the effect of adding the TSG on the thermal expansion rise of the composites, which would be crucial to adjust the pore size, was clearly demonstrated.

### 3.4. Effect of TSG on Air Permeability

**Figure 7:**
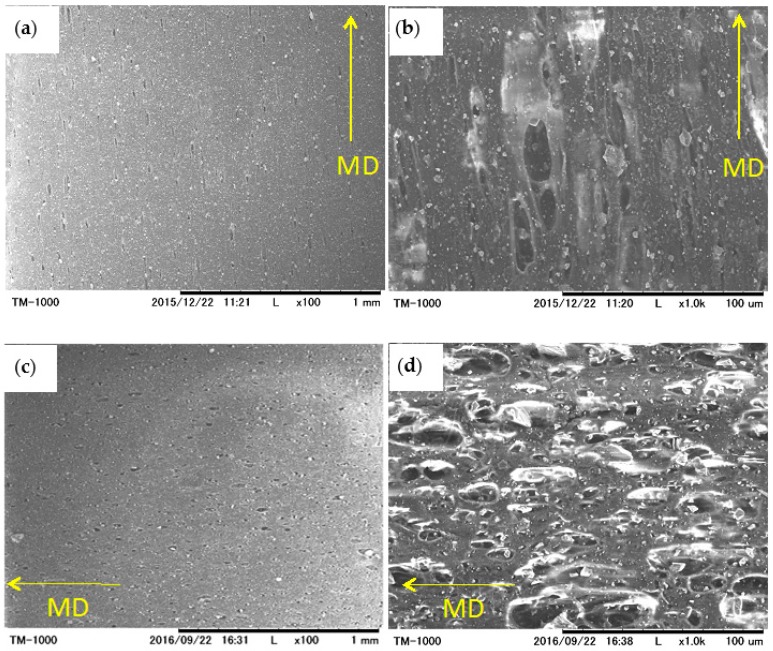
SEM micrographs of the surface for biaxially-oriented LLDPE/CaCO_3_ (**a**,**b**) and TSG/LLDPE/CaCO_3_ (**c**,**d**) films. MD represents machine direction; (**a**,**c**) are low-magnification pictures; and (**b**,**d**) are relatively high-magnification pictures.

The microporous structure of the prepared LLDPE/CaCO_3_ and TSG/LLDPE/CaCO_3_ composite films were observed by SEM. The film samples stretched 2.5 times biaxially were used and their surfaces were observed. The biaxial stretch forms pores, and the actually-used films in commercial products are the stretched films. In order to compare with the commercial LLDPE/CaCO_3_ films, the actual application condition of 2.5 times the biaxial stretch was used to prepare the SEM samples. Figure 7 shows their SEM micrographs. Long and narrow pores along machine direction (MD) were observed for non-TSG-added LLDPE/CaCO_3_ films. The length and width of pores along MD were 5–50 μm and 5–15 μm, respectively. Clearly, the pore enlargement in the transverse direction (TD) was limited. For TSG-added TSG/LLDPE/CaCO_3_ films, it was found that the limitation of pore enlargement on TD was attenuated. Pores with a uniform size of 5–40 μm on both MD and TD were well-formed probably due to the contribution of soft molecular chains of the TSG, benefitting from its lower melting temperature. According to the SEM observation results, the TSG showed a significant effect in pore size and shape adjustment. 

**Figure 8:**
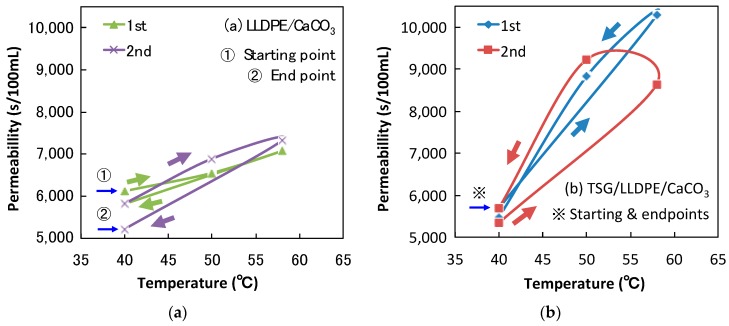
Temperature-permeability dependency of LLDPE/CaCO_3_ (**a**) and TSG/LLDPE/CaCO_3_ (**b**) composite films.

**Figure 9:**
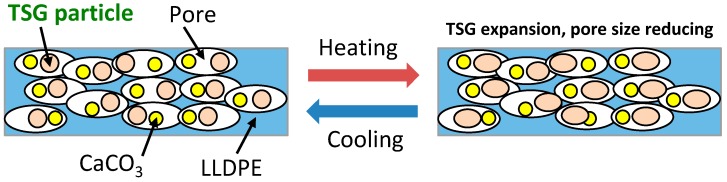
The working mechanism related to thermal expansion and pore size readjustment for the microporous TSG/LLDPE/CaCO_3_ composite films. As the temperature increases, TSG expands and, as a result, the pore size reduces. The pore size adjustment behavior is reversible in response to temperature.

To further explore the effect of TSG, the air permeability testing of LLDPE/CaCO_3_ and TSG/LLDPE/CaCO_3_ composite films was conducted. The temperature dependence of permeability for air transport through composite film samples with two heating/cooling cycles is presented in Figure 8. As Figure 8 shows, the testing temperature range is 40–58 °C. With an increase in temperature, permeation time per 100 mL air became longer for both LLDPE/CaCO_3_ (Figure 8a) and TSG/LLDPE/CaCO_3_ (Figure 8b) films. The difference in permeability of LLDPE/CaCO_3_ films at 40 and 58 °C was 900–2100 s/mL, while, for TSG/LLDPE/CaCO_3_ films, it was 2900–4600 s/mL, more than twice the value of the LLDPE/CaCO_3_ films. That is, the addition of TSG lengthened the permeation time more than two times when the temperature increased close to the melting point of the TSG. The first and second testing cycles showed a similar trend. Generally, a longer time corresponds to low air permeability, implying a smaller size of pores. This means the air permeability (pore size) adjustment function of the TSG works very well in TSG-added TSG/LLDPE/CaCO_3_ films. Figure 9 represents the working mechanism related to thermal expansion and pore size readjustment for the microporous TSG/LLDPE/CaCO3 composite films. During heating, the crystals of TSG near the pores melt and becomes a viscous liquid, thus, the volume increases to decrease the pore size. On the other hand, during cooling, the recrystallization of TSG crystals lowers the volume to increase the pore size once again. The pore size adjustment behavior is reversible in response to temperature. The controllable air permeability of microporous composite films was realized successfully by adding the TSG.

### 3.5. Effect of TSG on Temperature Control

**Figure 10:**
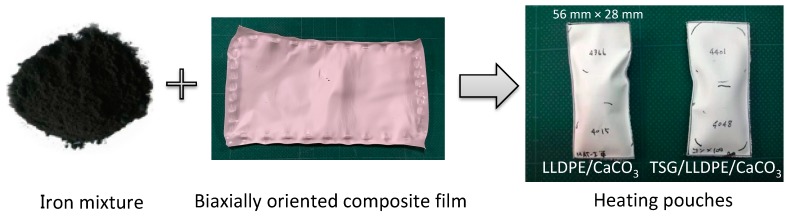
Hand-made heating pouches. The size of the pouches is 56 mm in length and 28 mm in width.

**Figure 11:**
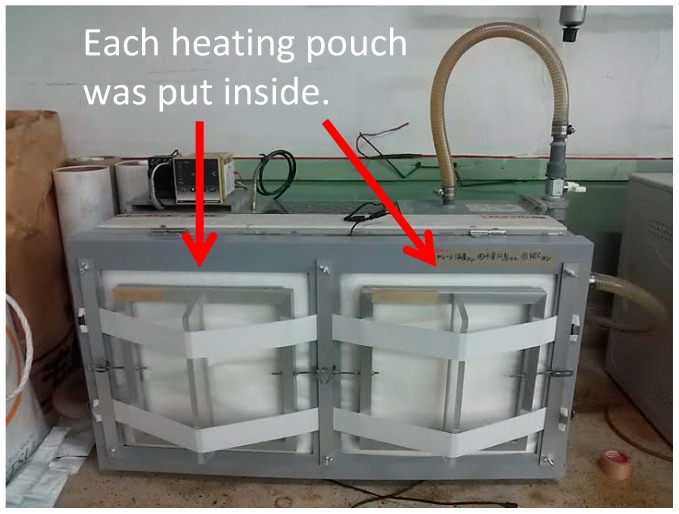
Temperature-measuring instrument. Each heating pouch was closely pressed against the temperature sensor plate inside the sealed box.

**Figure 12:**
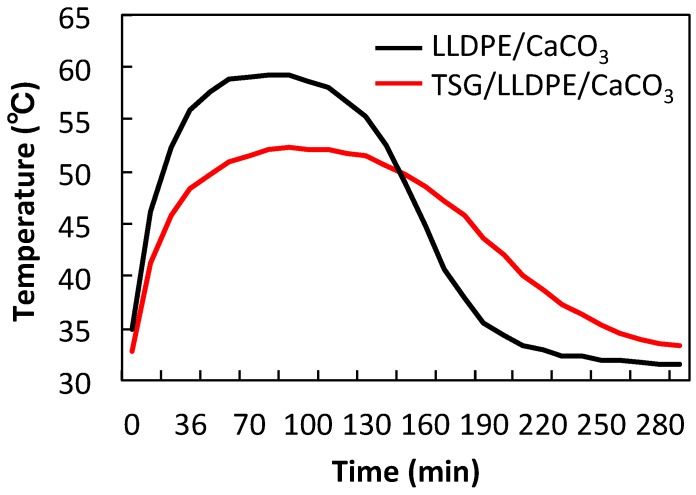
Temperature as a function of time for heating pouches of LLDPE/CaCO_3_ and TSG/LLDPE/CaCO_3_ composite films.

The demonstration temperature control experiments were further conducted to prove the pore-size adjustment effect of the TSG. The heating pouches made from LLDPE/CaCO_3_ and the TSG/LLDPE/CaCO_3_ composite films and iron mixture are shown in Figure 10. The temperature-measuring instrument is shown in Figure 11. The experiments were conducted from the ambient temperature. The detected temperature change with time was plotted and given in Figure 12. The maximum temperature of using LLDPE/CaCO_3_ was 59 °C, 7 °C higher than that of using TSG/LLDPE/CaCO_3_. After 150 min, the temperature holding was also better for using TSG/LLDPE/CaCO_3_. The temperature increase was mild from 40 °C, and the high temperature maintained a longer time when using TSG/LLDPE/CaCO_3_ composite films. This suggests less oxygen entered the pouch to react with the iron powder to generate heat, that is, the pore size reduced with the temperature increasing for the TSG/LLDPE/CaCO_3_ composite films. This is consistent with all the results mentioned above. The addition of TSG can give the composite films’ pore-size adjustment (controllable gas-permeability) function, and this was further proved to be feasible for practical applications by the demonstration temperature control experiments. 

## 4. Conclusions

In this work, we synthesized a novel TSG with good thermal durability and a lower melting temperature below 60 °C. TSG particles with a size of 10–400 μm were also produced successfully. The coefficient of linear thermal expansion (α) was as high as 2817.0 × 10^−5^/°C at 65–70 °C, and the excellent thermal expansion of TSG was revealed by TMA and PVT testing.

To effectively utilize the thermal expansion of the TSG, we proposed the usage of TSG particles to reduce the pore size to adjust the gas permeability for breathable films under temperature simulation. By introducing the TSG into LLDPE/CaCO_3_, we prepared the microporous TSG/LLDPE/CaCO_3_ composite films. The TSG/LLDPE/CaCO_3_ composite films had well-balanced mechanical properties, their thermal expansion behavior was also clarified, according to the tensile testing and TMA results.

The SEM micrographs indicated the uniformly-porous structure and the pore size was in the range of 5–40 μm for the TSG/LLDPE/CaCO_3_ composite films. The addition of TSG lengthened the permeation time almost two time at around 60 °C, showing the pore size reduced due to the thermal expansion effect of the TSG near and above the melting temperature. Furthermore, to verify the practical feasibility of the functional TSG/LLDPE/CaCO_3_ composite films, we conducted the demonstration temperature control experiments. The experimental results clearly proved the addition of TSG offered the effect of pore-size adjustment (controllable gas-permeability), more promisingly, the great practical value and application prospects of this strategic effect for a temperature-sensitive breathable film was clarified.

The global functional porous membranes/films market is expected to grow during the forecast period [36]. The major drivers of this growth will be the increasing awareness and concern for hygiene and healthy life. With the increase in the food and beverage industry, the packaging industry will also be affected. The breathable films for the packaging industry to maintain the quality and freshness of the food items during the shelf period is also a quite promising application field of TSG/LLDPE/CaCO_3_ films, because we are able to provide a humidity adjustment function readily at the same time with the microporous films.

## Figures and Tables

**Table 1:** The coefficient of linear thermal expansion (α) at various temperature intervals obtained by TMA measurement for TSG films.

Sample	Coefficient of Linear Thermal Expansion, α (10^−5^/°C)		
	10–50 °C	55–65 °C	65–70 °C
TSG	4.5	322.2	2817.0

^1^ The initial specimen length L_0_ is 23308.3 μm at 10 °C.

**Table 2:** The volumes and change rates of volume for TSG and LLDPE/CaCO_3_ samples measured by the PVT test.

Samples	Volume (cm^3^)		Change Rate of Volume (%)
	30 °C	60 °C	30–60 °C
TSG	0.443	0.459	3.530
LLDPE/CaCO_3_	0.391	0.395	1.111

**Table 3:** Mechanical properties of LLDPE/CaCO_3_ and TSG/LLDPE/CaCO_3_ composites.

Samples	Tensile Strength at Break (MPa)	Young’s Modulus (MPa)	Elongation (%)
LLDPE/CaCO_3_	39 ± 2	147.7 ± 0.3	571 ± 5
TSG /LLDPE/CaCO_3_	28.32 ± 2	145.5 ± 0.5	699 ± 5

## References

[1] Gong J., Shitara M., Serizawa R., Makino M., Kabir M.H., Furukawa H. (2014). 3D printing of meso-decorated gels and foods. Mater. Sci. Forum.

[2] Gong J., Igarashi S., Sawamura K., Furukawa H. (2013). Gel engineering materials meso-decorated with polymorphic crystals. Adv. Mater. Res..

[3] Gong J., Watanabe Y., Watanabe Y., Hidema R., Kabir M.H., Furukawa H. (2013). Development of a novel standard type of gel engineering materials via simple bulk polymerization. J. Solid Mech. Mater. Eng..

[4] Kabir M.H., Gong J., Watanabe Y., Makino M., Furukawa H. (2013). Hard-to-soft transition of transparent shape memory gels and the first observation of their critical temperature studied with scanning microscopic light scattering. Mater. Lett..

[5] Gong J., Furukawa H. (2013). Smart optical device of varifocal lens developed with high transparent shape memory gels. Expect. Mater. Future.

[6] Amano Y., Hidema R., Gong J., Furukawa H. (2012). Creation of shape memory gels with inter-crosslinking network structure. Chem. Lett..

[7] Harada S., Hidema R., Gong J., Furukawa H. (2012). Intelligent button developed with smart soft and wet materials. Chem. Lett..

[8] Kwon Y.M., Kim S.W., Kwon G.S. (2005). Thermosensitive biodegradable hydrogels for the delivery of therapeutic agents. Drugs and the Pharmaceutical Sciences, Polymer Drug Delivery System.

[9] Chandra A., Meyer W.H., Best A., Hanewald A., Wegner G. (2007). Modifying thermal expansion of polymer composites by blending with a negative thermal expansion material. Macromol. Mater. Eng..

[10] Takenaka K., Ichigo M. (2014). Thermal expansion adjustable polymer matrix composites with giant negative thermal expansion filler. Compos. Sci. Technol..

[11] Poveda R.L., Gupta N. (2016). Thermal expansion of CNF/polymer composites. Carbon Nanofiber Reinforced Polymer Composites.

[12] Kiba S., Suzuki N., Okawauchi Y., Yamauchi Y. (2010). Prototype of low thermal expansion materials: Fabrication of mesoporous silica/polymer composites with densely filled polymer inside mesopore space. Chem. Asian J..

[13] Yamashina N., Isobe T., Ando S. (2012). Low Thermal Expansion Composites Prepared from Polyimide and ZrW2O8 Particles with Negative Thermal Expansion. J. Photopolym. Sci. Technol..

[14] Wei C., Srivastava D., Cho K. (2002). Thermal Expansion and Diffusion Coefficients of Carbon Nanotube-Polymer Composites. Nano Lett..

[15] Nishino T., Kotera M., Sugiura Y. (2009). Residual stress of particulate polymer composites with reduced thermal expansion. Journal of Physics: Conference Series.

[16] Sideridou I., Achilias D.S., Kyrikou E. (2004). Thermal expansion characteristics of light-cured dental resins and resin composites. Biomaterials.

[17] Yang B., Yang W. (2003). Thermo-sensitive switching membranes regulated by pore-covering polymer brushes. J. Membr. Sci..

[18] Albo J., Wang J., Tsuru T. (2014). Gas transport properties of interfacially polymerized polyamide composite membranes under different pre-treatments and temperatures. J. Membr. Sci..

[19] Pilar Castejón P., Habibi K., Saffar A., Ajji A., Martínez A.B., Arencón D. (2018). Polypropylene-Based Porous Membranes: Influence of Polymer Composition, Extrusion Draw Ratio and Uniaxial Strain. Polymers.

[20] Park Y., Gutierrez M.P., Lee L.P. (2016). Reversible self-actuated thermo-responsive pore membrane. Sci. Rep..

[21] Sershen S.R., Westcott S.L., Halas N.J., West J.L. (2000). Temperature-sensitive polymer-nanoshell composites for photothermally modulated drug delivery. J. Biomed. Mater. Res. Part A.

[22] He Y., Moreira E., Overson A., Nakamura S.H., Bider C., Briscoe J.F. (2000). Thermal characterization of an epoxy-based underfill material for flip chip packaging. Thermochim. Acta.

[23] Choi Y.J., Yamaguchi T., Nakao S.I. (2000). A novel separation system using porous thermosensitive membranes. Ind. Eng. Chem. Res..

[24] Park Y.S., Ito Y., Imanishi Y. (1998). Permeation control through porous membranes immobilized with thermosensitive polymer. Langmuir.

[25] Shtanko N.I., Kabano V.Y., Apel P.Y., Yoshida M. (1999). The use of radiation-induced graft polymerization for modification of polymer track membranes. Nucl. Instrum. Methods Phys. Res. Sect. B.

[26] Chung D.J., Ito Y., Imanishi Y. (1994). Preparation of porous membranes grafted with poly (spiropyran-containing methacrylate) and photocontrol of permeability. J. Appl. Polymer Sci..

[27] Iwata H., Oodate M., Uyama Y., Amemiya H., Ikada Y. (1991). Preparation of temperature-sensitive membranes by graft polymerization onto a porous membrane. J. Membr. Sci..

[28] Hotpress Gilfillan W.N., Moghaddam L., Bartley J., Doherty W.O.S. (2015). Thermal extrusion of starch film with alcohol. J. Food Eng..

[29] Ruland W. (1961). X-ray determination of crystalinity and diffuse disorder scattering. Acta Crystallogr..

[30] Roger B. (1999). Handbook of Polymer Testing—Physical Methods.

[31] Landsberg M.I., Winston G. (1947). Relationship between measurements of air Permeability by two machines. Text. Res. J..

[32] Chakravorty S. (2002). PVT testing of polymers under industrial processing conditions. Polym. Test..

[33] Huang D.H., Tran T.N., Yang B. (2014). Investigation on the reaction of iron powder mixture as a portable heat source for thermoelectric power generators. J. Therm. Anal. Calorim..

[34] Mo Z., Zhang H. (1995). The degree of crystallinity in polymers by wide-angle x-ray diffraction (WAXD). J. Macromol. Sci. Part C Polym. Rev..

[35] Hosaka E., Gong J., Ito H., Shibata Y., Nakanishi D., Ishihara S. Processing and Thermal Response Properties of Temperature Sensitive Gel/Polymer composites. Design, Manufacturing and Applications of Composites. Proceedings of the Eleventh Joint Canada-Japan Workshop on Composites: First Joint Canada-Japan-Vietnam Workshop on Composites.

[36] Technavio (2017). Top 5 Vendors in the Breathable Films Market from 2017 to 2021.

